# Onboard Evaluation of Variable Water Flow and Recirculation Effects on Bleeding of Atlantic Cod (*Gadus morhua*)

**DOI:** 10.3390/foods9111519

**Published:** 2020-10-22

**Authors:** Saemundur Eliasson, Sigurjon Arason, Bjorn Margeirsson, Olafur P. Palsson

**Affiliations:** 1 Faculty of Industrial Engineering, Mechanical Engineering and Computer Science, University of Iceland, Hjardarhagi 2-6, IS-107 Reykjavik, Iceland; bjorn.margeirsson@saeplast.com (B.M.); opp@hi.is (O.P.P.); 2 Matis (Icelandic Food and Biotech R&D), Vinlandsleid 12, IS-113 Reykjavik, Iceland; sigurjar@hi.is; 3 Faculty of Food Science and Nutrition, University of Iceland, Eiriksgata 29, IS-101 Reykjavik, Iceland; 4 Saeplast Iceland, Gunnarsbraut 12, IS-620 Dalvik, Iceland

**Keywords:** Atlantic cod, bleeding, water flow, recirculation, fatty acids, lipids, heme iron

## Abstract

The aim of the study was to explore the effects of different design variables in the onboard bleeding process of cod on bleeding efficiency and the resulting product quality. A time- and flow-controlled process was used to create variable bleeding conditions for whole gutted cod onboard a wet-fish trawler. Two main design variables influencing the bleeding process are the pump flow recirculation (PFR) and the water replacement ratio (WRR); they were studied in five different combinations (groups). The effects of different bleeding conditions were evaluated by measurements of free fatty acids (FFAs), phospholipids (PLs), and total heme iron (HI) content during freezer storage for up to four months. The results for PL content and the regression model indicate that the enzyme activity in the fish muscle is lower in cases where PFR exerts greater influence in the bleeding process than WRR. The effects of successful blood removal also seem to be most noticeable after one month of freezer storage, rather than in fresh cod after seven days or after four months of simulated frozen food-chain storage. The study indicates that, with the bleeding medium to fish ratio of around 3:1 and enough WRR (over 100% replacement in 20 min), the PFR becomes the limiting design parameter regarding efficient blood removal and should be at least 10% of the tank volume per minute to ensure enough recirculation and flow of water in the bleed-out tanks.

## 1. Introduction

An increasing demand for high-quality fresh fish has led fish producers globally to focus on and improve raw material handling and processing. Improvements in temperature control and packaging have extended the shelf-life of fish and promoted an increase in fresh fish processing and export [1,2,3,4]. High-end products, like fresh cod loins, also require the raw material to be well drained of blood and uniform in color. The present experiment was conducted onboard a wet-fish trawler with newly developed time- and temperature-controlled bleeding and superchilling equipment.

Bleeding is generally the first process the fish goes through after catching and is aimed to drain most of the blood from the muscle [5]. Depending on the species, blood makes up about 1.5–7% of a fish’s total body weight with around 20% of it localized in the muscular tissue [6]. Insufficient bleeding can affect the shelf-life of products through product taste, odor, visual appearance, and residual blood, which can also promote lipid oxidation, provide nourishment for bacteria, and cause increased enzymatic activity [7].

Botta et al. [8] demonstrated in their research that the bleeding process of the fish is an important factor for improving the color of the flesh. Cutting the isthmus and gutting in a one-step procedure, where the isthmus is cut without touching the backbone and then the fish is immediately gutted, is the most common practice in fresh fish processing. Studies [8,9,10] have indicated that the time-factor was a more important aspect of the procedure of the bleeding process than the cutting method itself. Others have studied the influence of stress [11,12,13], time [9,10,14], and temperature [10,15,16] on the bleeding process. Karlsdottir et al. [16] concluded that if myoglobin and hemoglobin proteins remain in the white muscle tissue, the effect of an improper exsanguination can cause increased rancidity of the product and shorten the shelf-life.

Lipid oxidation is one of the major problems associated with seafood during processing and storage [17]. Typical oxidative response by muscle foods is variable, depending on processing treatment, and freezing generally inhibits the oxidative response [18]. Research has shown that when phospholipids (PLs) are degraded due to enzymatic activity, the amount of free fatty acids (FFAs) increases [16,19]. Measurements of the amount of free fatty acids (FFAs) and phospholipids (PLs) can therefore be a good indicator of quality when working with lean fish. Several variables can impact the amount of enzymatic activity in fish, including freezing rate, storage temperature, storage time, temperature fluctuations during storage, and bleeding efficiency [16,19]. Intact lipids, FFAs, and oxidized lipids can interact with proteins and result in quality deterioration of lean fish species. Results by Burgaard and Jorgensen [20] and Dang et al. [21] show how changes in lipids during frozen storage of fish can lead to quality deterioration, especially at temperatures around −20 °C to −10 °C. Temperature variations during transportation and short-term freezer storage commonly cause fish temperatures to reach above −20 °C, resulting in negative changes in quality [21,22].

Blood components in the fish muscle have been proven to contribute to the lipid oxidation of fish products during processing and storage. Also, the iron ions released from heme proteins are thought to promote lipid oxidation [7]. Heme proteins, including hemoglobin (Hb) and heme iron (HI), play an important role in lipid oxidation as pro-oxidants [23]. Phospholipids account for almost half of the total lipids in the blood of fish [24], and it has been suggested that they contribute to lipid oxidation of fish muscle, since phospholipids are generally known to be highly susceptible towards oxidation [25].

Conditions in industrial bleed-out tanks that influence the bleeding efficiency are mainly the water replacement ratio (WRR), which is the ratio of bleeding medium replaced (seawater in onboard systems), and the pump flow recirculation (PFR), which impacts the flow turbulence in the tanks. The influence of the WRR and PFR on the bleeding process of cod has, according to the authors’ best knowledge, not been studied further. The aim of this research is to explore the effects of WRR and PFR of the bleeding medium on the bleeding efficiency (exsanguination) of Atlantic cod (*Gadus morhua*) and the resulting product quality by evaluating FFA, PL, and HI content.

## 2. Materials and Methods

The experiments took place during a fishing trip where cod from the same haul was bled for 20 min in five different conditions (groups) but otherwise handled in the same way. The cod was then processed at a fish processing plant in Saudarkrokur, Iceland, four days after the catch, and the fillets were kept frozen and measurements were made at Matis (Icelandic Food and Biotech R&D) in Reykjavik, Iceland. The measurements were done after three storage times: on fresh cod seven days from catch, then frozen after 1 month and 4 months. As the general industry standard is variable, the samples were stored at a relatively high freezer storage temperature (−12 to −18 °C) during the last period of frozen storage to presumably result in a more rapid quality decline (PLs and FFAs), simulating imperfect and realistic temperature control in transport and long-term freezer storage [21].

### 2.1. Experimental Design

**Figure 1:**
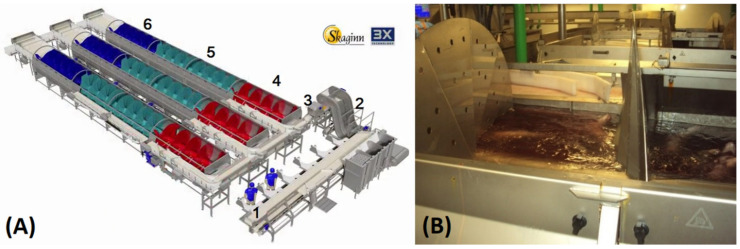
(**A**) Three-dimensional model of screw conveyor used for controlled bleeding and superchilling. Figure labels: No.1: Gutting; No.2: Washing; No.3: Grading; No.4: Bleeding; No.5: Chilling; No.6: Superchilling [23]. (**B**) Photo of screw conveyor used for controlled bleeding.

The cod for the experiment was caught by the wet-fish trawler Malmey SK-1 (no. 1833) on 4 March 2016, north west of Iceland in a sea temperature of 5–6 °C. The cod came from a haul of 5.3 tons with a tow-time of 90 min—the haul size was within the company guidelines of around 7 tons and the tow-time was below average for the fishing trip. After gutting all samples alive (within 30 min after hauling), cutting the isthmus and gutting in a one-step procedure, they were placed under different bleeding conditions with regard to recirculation and flow rate but otherwise handled and stored the same way. The temperature of the seawater in the bleed-out tanks was 5 °C. Groups A, B, and C were bled for 20 min in time-controlled bleed-out tanks equipped with a screw conveyor (manufactured by Skaginn 3X, Akranes, Iceland [26], see Figure 1A,B). The screw conveyor is basically an Archimedes screw that transfers the gutted fish through the bleed-out tanks in chamber batches. The volume capacity of all the bleed-out tanks is about 7 m^3^, and each of the ten slots of the bleed-out tank system holds about 150 kg of cod at a time.

The power of the recirculating pump and recirculation rate in the tank were varied between the groups; the different settings between the bleeding systems are shown in Table 1. The table shows the conditions for each group during the bleeding procedure. The cod in Groups D and E were bled for 20 min in separate 460-L tubs, and the water was replaced in one batch after 10 min during the bleeding procedure. Each tub contained around 100 kg of cod and 200 kg of seawater. In one of the tubs (Group D), a pump was installed to recirculate the seawater, while another (Group E) had no pump (still seawater).

After the bleeding process, the samples (whole gutted fish) from each group were superchilled with chilled seawater/saltwater in a screw conveyor with a two-stage cooling process: first at −0.8 °C for 20 min and then at −2.8 °C for 8 min. The seawater in the chilling process also has the effect of surface cleaning and washing the fish after the bleeding. The samples were then packed in 460-L insulated tubs with no ice and stored in the ship hold at −1 °C for 40 h (tubs without lids and without drain plugs). Then 20 fish from each group were headed, filleted, and skinned and then manually trimmed. The fillets were then transported in 5-kg expanded polystyrene (EPS) boxes (manufactured by Tempra, Hafnarfjordur, Iceland) at 0 °C. Upon arrival to the laboratory, the fillets were kept at 0 °C storage for two days before being analyzed (on day seven from catch). For each sampling point, measurements were performed on three fillets from each group, and duplicate samples were measured from each fillet (*n* = 6). The rest of the fillets were frozen down to −24 °C in a blast freezer (20T 2/1 POS/NEG REMO, Ilsa Spa, San Fior, Italy). They were stored at −24 °C for four weeks prior to analysis, the one-month sampling point, where three fillets from each group were measured. Subsequently, the remaining samples were transported directly (no thawing) to a −12 °C storage for six weeks followed by −18 °C for eight weeks before analysis. These variations of common storage temperature at −18 °C and high storage temperature at −12 °C were selected to simulate the rapid quality deterioration during an uncontrolled transport and storage in the value chain of frozen fillets. The frozen samples were measured after simulated food-chain freezer storage (six weeks at −12 °C followed by eight weeks at −18 °C).

### 2.2. Chemical Analyses

Fillet samples were taken from a freezer storage at −24 °C and thawed in air at 0 °C for about 48 h on a metal frame, covered with a plastic sheet to prevent drying. Mid-section fillet parts were used for measurements of fat, FFAs, and PLs, but unbound and bound iron, water content, and salt were measured in all parts of the fillet samples. Total fat content in the muscle was measured using extraction of total lipids [27]. This extraction was then used to estimate the amount of FFAs and the proportion of PLs in the fat measured. The method used for determination of FFA content was the method from Lowry and Tinsley [28] with modification by Bernardez et al. [29]. The procedure of the modified method was to use duplicate samples in two screw cap glass tubes with the removed solvent from the lipid extraction [27]. Then 3 mL of cyclohexane and 1 mL of cupric acetate-pyridine reagent were added, with contact time >30 s. The mixture was vortexed for 40 s and centrifuged at 2000× *g* for 10 min at 4 °C (Heraeus Biofuge Stratos, Thermo Scientific, Bremen, Germany). The upper layer of the sample was read at 710 nm (Amersham Pharmacia Biotech, Ultrospec 3000 pro, Buckinghamshire, United Kingdom). Quantification was based on a calibration curve constructed from oleic acid standards and the FFA content was calculated by the following equation [29] (oleic acid in Equation (1) stands for µmol of oleic acid and 282.46 is its molecular weight):

(1) $$\mathrm{FFA}:\;\mathrm{free}\;\mathrm{fatty}\;\mathrm{acid}\;\mathrm{content}\;(\%)\;=\;\frac{\mathrm{oleic}\;\mathrm{acid}\;\times \;282.46\;M\;\times \;1*10-6}{g\;\mathrm{lipid}\;\mathrm{in}\;\mathrm{the}\;\mathrm{sample}}\;\times \;100$$

The method used for the estimation of PL content was the colorimetric method based on the formation of a complex between phospholipids and ammonium ferrothiocyanate [30]. Duplicates were made of each extract from the lipid extraction. First, 2 mL of chloroform was added to a 15-mL plastic tube with a screw cap. Next, 10 µL of the lipid extract was then added to the tube as well as 1 mL of thiocyanate reagent. The mixture was vortexed for 1 min and centrifuged at 2000 rpm for 5 min at 4 °C. (Beckman Coulter Inc., TJ-25 Centrifuge, Indianapolis, IN, USA). The lower layer was read at 480 nm and compared with known amounts of a standard phospholipid solution.

The HI (heme iron) content was determined according to the method described by Gomez-Basauri and Regenstein [31] with the main reagent being 40 mM phosphate buffer at pH 6.8 (disodium hydrogen phosphate). A 2-g grounded sample was weighed into a 50-mL centrifuge tube and 20 mL of cold 40 mM phosphate buffer was added. The content was homogenized at 13.500 rpm for 10 sec and then centrifuged at 3000× *g* for 30 min at 4 °C (Beckman Coulter Inc., TJ-25 Centrifuge, Indianapolis, IN, USA). The supernatant was filtered using Whatman No. 1 filter paper and then the filtrate read at 525 nm (Amersham Pharmacia Biotech, Ultrospec 3000 pro, Buckinghamshire, United Kingdom). Myoglobin content was calculated from the millimolar extinction coefficient 7.6 and a molecular weight of 16.110. The HI content was calculated based on myoglobin, which contains 0.35% iron, and expressed as mg/100 g sample.

### 2.3. Statistical Analyses

SigmaStat v. 3.5 for Windows (Systat Software, Inc., San Jose, CA, USA) and Microsoft Excel 2016 (Microsoft Inc. Redmond, WA, USA) were used for statistical analysis. One-way analysis of variance (ANOVA) and Duncan’s post hoc test were applied for evaluating significance between groups. During analysis, a difference at the level of *p* < 0.05 was considered statistically significant. In order to evaluate the results for the five different groups, a multivariable linear regression model shown in Equations (2)–(4) was constructed [32]:

(2) $$FFA=b_{0}+b_{1}PFR+b_{2}\;WRR+\varepsilon$$

(3) $$PL=b_{0}+b_{1}PFR+b_{2}\;WRR+\varepsilon$$

(4) $$HI=b_{0}+b_{1}PFR+b_{2}\;WRR+\varepsilon$$

where FFA, PL, and HI are dependent variables, and PFR and WRR are independent variables, $b_{i}$ represents the model parameters and ε is the error or residuals (with mean 0 and variance $\sigma^{2}$).

The objectives of the regression model are to evaluate the relation between the dependent variables, FFA, PL, and HI, on one hand and the effects of the independent variables, PFR and WRR, on the other hand. The parameters in Equations (2)–(4) are estimated in SigmaStat based on the total available observations.

## 3. Results

**Figure 2:**
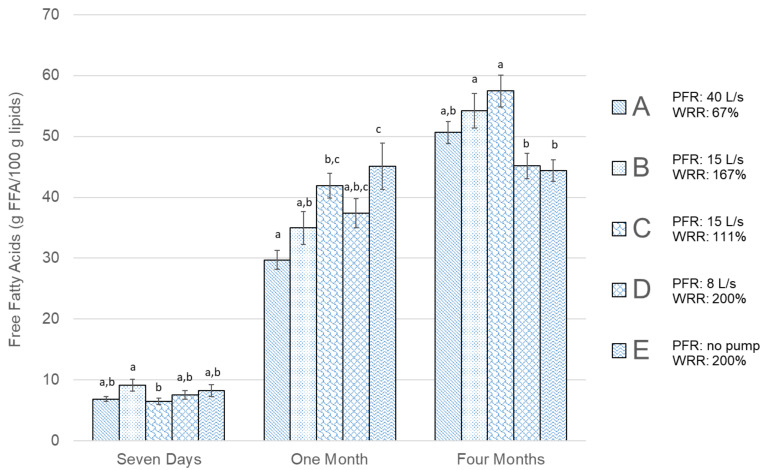
Free fatty acids (g FFA/100 g lipids) results for all groups shown in Table 1. The error bars show the standard error of the mean values ($\bar{\mu }\pm \sigma_{\bar{\mu }}$), *n* = 6. Different letters (a, b, and c) within storage times represent significant differences between experimental groups (*p* < 0.05).

**Figure 3:**
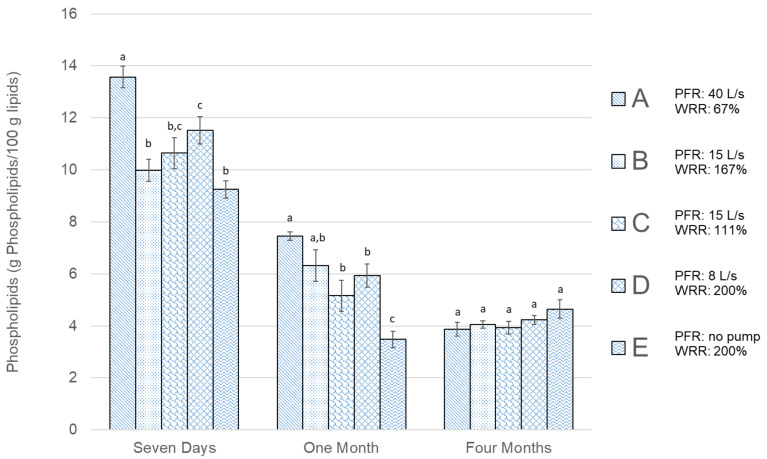
Phospholipids (g PL/100 g lipids) results for all groups shown in Table 1. The error bars show the standard error of the mean values ($\bar{\mu }\pm \sigma_{\bar{\mu }}$), *n* = 6. Different letters (a, b, and c) within storage times represent significant differences between experimental groups (*p* < 0.05).

**Figure 4:**
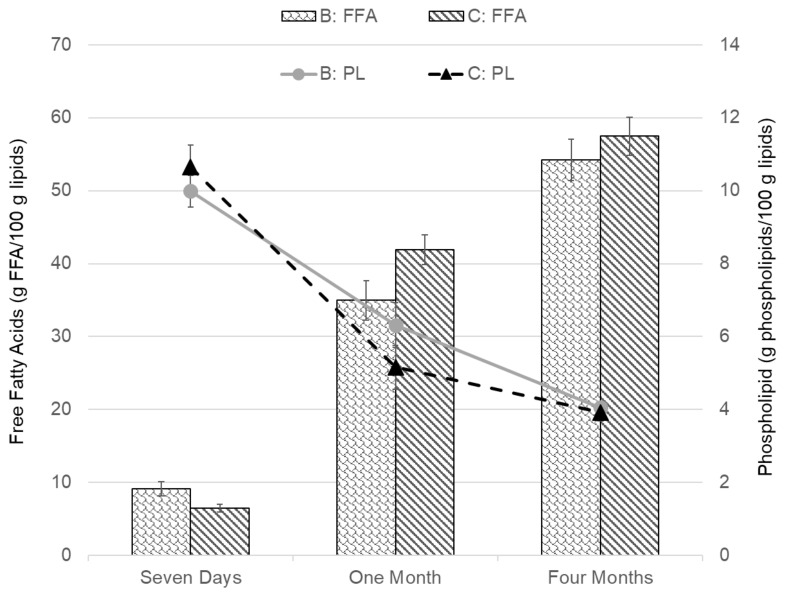
Comparison of Free Fatty Acids (FFA) and Phospholipids (PL) results for Groups B and C. The error bars show the standard error of the mean values ($\bar{\mu }\pm \sigma_{\bar{\mu }}$).

**Figure 5:**
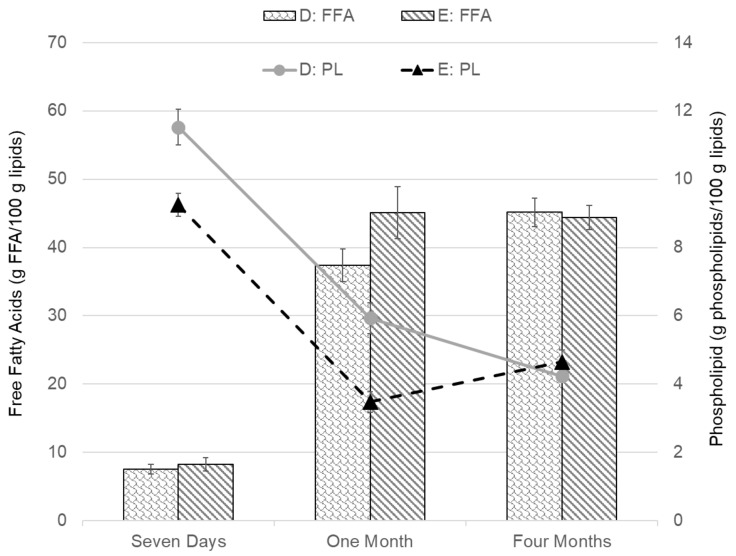
Comparison of Free Fatty Acids (FFA) and Phospholipids (PL) results for Groups D and E. The error bars show the standard error of the mean values ($\bar{\mu }\pm \sigma_{\bar{\mu }}$).

**Figure 6:**
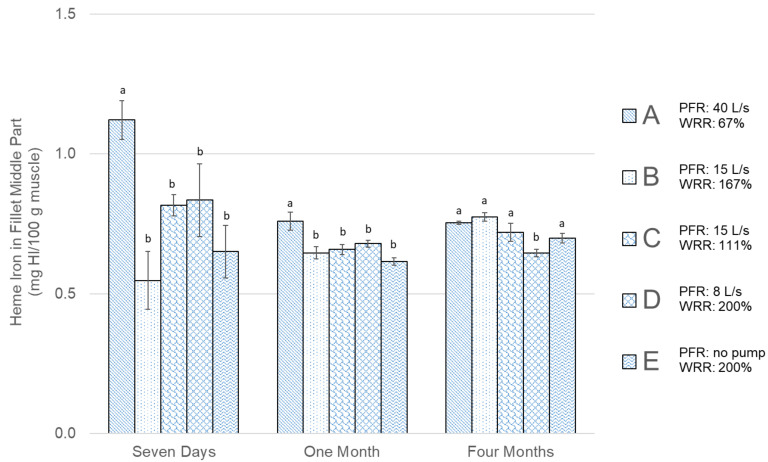
Heme Iron (g HI/100g muscle) results for all groups shown in Table 1. The error bars show the standard error of the mean values ($\bar{\mu }\pm \sigma_{\bar{\mu }}$), *n* = 6. Different letters (a and b) within storage times represent significant differences between experimental groups (*p* < 0.05).

The results of the measurements of FFA content in Figure 2 and PL content in Figure 3 show that prolonged frozen storage affects the amount of both FFAs and PLs negatively (i.e., the FFA content increases with longer storage time, while the PL content is reduced). The values in Figure 2, Figure 3, Figure 4, Figure 5 and Figure 6 are presented as mean values ± standard error of the mean. The values of FFAs (Figure 2) in the fresh cod samples after seven days show a significant difference between Group B and Group C and no significant difference for other groups. After one month of frozen storage, Figure 2 shows that FFA was higher in all groups and there was a linear trend between the three groups bled in the screw conveyor tanks (A, B, and C). Significantly higher FFA content was measured in Group E compared to the other groups after one month of frozen storage. All groups except E showed a rise in FFA content between months one and four. The FFA trend for Groups A, B, and C was similar between one and four months, while the difference was relatively smaller for Group D and E. The PL content results in Figure 3 show that Group A had significantly the highest PL content in fresh cod after seven days of storage. After freezing, and as the storage time became longer, there was a decline in PL content for all groups, except Group E, which showed lower PL values after one month of freezer storage compared to four months. Therefore, after one month, Group E showed significantly different results compared to the other groups, showing both the highest FFA content and the lowest PL content. After four months of frozen storage, there was no significant difference in PL content between the groups.

The variables PFR and WRR (parameters shown in Table 1) can influence the difference between groups in more than one way. For Group A, both variables are set with different parameters from the other groups. As PFR is comparable between Groups B and C, and WRR is comparable between Groups D and E, the same FFA and PL content results are shown together in Figure 4 and Figure 5. The FFA and PL content of Groups B and C, for which the PFR is the same but the is WRR different, are shown and compared in Figure 4. The greatest difference between the groups was after one month of freezer storage; however, the difference there was determined to be insignificant (*p* > 0.05). Looking at a comparison between Groups D and E in Figure 5, where the WRR is the same but the PFR is different, a significant difference in FFA content was not observed between the groups. The PL values were, however, significantly different between the groups, both after seven days and one month of frozen storage, showing higher PL content for Group D. However, after four months of storage, there was no significant difference between Groups D and E.

Figure 6 shows the measurement results of HI content for samples of unfrozen fillets seven days from catch and fillets stored frozen from one month at −24 °C up to four months in a freezer storage ranging from −12 °C to −18 °C. After seven days, the highest HI values in fresh cod were in Group A, which were significantly different from other groups, which did not show a significant difference between them. After one month of freezer storage, the trend was the same, with significantly higher HI in Group A compared to the other groups. After four months, the difference in HI values was negligible between groups, except for Group D, which showed significantly lower HI content compared to the other groups.

The results of the regression model are shown in Table 2. For each of the dependent variables (FFA, PL, and HI), the model parameters (b_0_, b_1_, and b_2_) and their significance are shown. The overall model significance, Prob(F), and *R*^2^ values are also listed for dependent variables. The model results show that the model parameters are mostly significant after one month of freezer storage for variables FFA and PL, where all parameters except one are significant to *p* < 0.05. For the variable HI and the seven-day and four-month time periods for all dependent variables, fewer significant parameters were found.

## 4. Discussion

The design of this study was industry based, as it was performed onboard a trawler during fishing, and is consequently difficult to repeat in full detail due to variability. This makes the study unique but also presents the challenge of isolating the specific variables of interest, PFR and WRR. Studies show that the residual blood of bled fish can be substantial [6,7,13,14]. The bleeding process in this study was designed to provide optimal bleeding conditions with regard to other factors previously studied [5,33,34,35]. The fish were also bled alive within 30 min, as suggested by Olsen et al. [9], and a washing time of 20 min was ensured [10,14]. While the subject of the bleeding of cod has been studied with regard to optimum bleeding time and bleeding methods [5,9,10,16], few have attempted to evaluate the effect of PFR and WRR, which are important design parameters. This study aimed to evaluate the effects of these variables at three different storage times based on the FFA, PL, and HI contents of cod fillets. The storage times studied were chosen to represent the effects of variable bleeding conditions on fresh cod (iced at 0 °C) seven days after catch, then on the freezing quality (quick frozen and stored at −24 °C), and, lastly, on a simulated frozen storage food-chain (between −12 and −18 °C). The simulated storage temperature used can be compared to industrial frozen storage studied by Dang et al. [21], where similar patterns for FFA and PL content in catfish were observed during the frozen storage period. Most studies on the bleeding of cod use residual blood measurements or some form of color evaluation [9,13,35] to assess the resulting effects. In this study, the reason for evaluating the bleeding using FFA, PL, and HI content is because of their relation to the enzyme activity in the muscle at different storage temperatures [16,36]. The results of this study are mostly directly comparable with the findings of Minh et al. [37,38] and Karlsdottir et al. [16], who also applied similar methods to investigate the bleeding of cod and other lean fish. Other studies using FFA, PL, and HI content as measurement references to bleeding efficiency and storage apply to other fish spices (e.g., cobia, seabass, tilapia, and catfish [21,38,39,40]).

The results showing FFA and PL development during simulated frozen storage in this study support the contribution of blood components in the fish to lipid oxidation [7]. The trend of FFA formation is similar to what others [16,21] have seen during frozen storage of cod with different bleeding processes, however, the FFA values in this study were relatively higher. The strong connection linking HI to susceptibility of lipid oxidation in the fish muscle [31] was less noticeable in the current study. The measured FFA content was generally low in fresh cod after seven days and similar to values for fresh cod that others have measured [37]. Some patterns were observed, indicating that during frozen storage the FFA content increased at different rates within the fish muscle depending on the group. The results shown in Figure 2 indicated that for groups bled in a screw conveyor, FFA was formed at a slower rate in Group A, which had the largest PFR but lowest WRR. However, after four months of simulated frozen food-chain storage, Groups D and E (which bled out in 460-L tubs with higher WRR) showed significantly lower values of FFA content compared to Groups B and C. This indicates the positive effects of higher values for both PFR and WRR. The FFA results in Figure 2 also show that through the storage period there was relatively less increment in FFA in Groups D and E that were bled in tubs, where PFR was low and WRR was high, compared to groups bled in a screw conveyor with lower WRR.

As suggested by Hardy et al. [41], a decrease in the PL content of lean fish like cod during frozen storage is the main factor driving the accumulation of FFA. A lower rise in FFA formation for Groups D and E indicate less enzyme activity (and slower PL formation) in fillets from those groups, indicating more effective blood removal. The results of total HI content (Figure 6) in the samples showed a significantly higher value for Group A for fresh cod after seven days and also after one month of freezer storage. After four months, there was relatively little difference between groups, with only Group D resulting in significantly lower HI content. The results of HI after seven days and one month, however, indicate that there were some negative effects relating to low WRR.

The PL content results in Figure 3 show a reverse pattern compared to the FFA content, as they decreased as storage time passed, due to enzyme activity [42]. After seven days, the highest significant PL value was for Group A, which had the highest PFR and the lowest WRR. The second highest significant PL content after seven days was in Group D, with relatively low PFR but high WRR. This indicates that for fresh cod, positive effects of both high PFR and WRR can be seen in the corresponding PL content. The lowest PL value average was for Groups E, with the lowest PFR and the highest WRR, however, it was not significantly lower than Groups B and C. The quantity of PL measured in fresh cod was relatively low, compared to the results of Minh et al. [37], although the sampling methods and handling were similar. After one month of freezer storage, these Groups, A and E, still showed the lowest and highest average in PL values among the five groups, with Group E significantly lowest. This indicates that blood removal was more effective in Group A, as PL values support that there was less enzyme activity compared to the other groups. However, after one month freezer storage, Group E showed both the significantly highest FFA and the lowest PL content, indicating negative effects of no pump/PFR even though the WRR was set relatively high. After four months of freezer storage, the PL values for all groups seemed to even out at around 4%, with no significant difference between groups. The FFA content for the corresponding period indicates that the increase in FFA originated from another source than decomposing PLs [21,42].

Comparing specifically Groups B and C (in Figure 4), where the WRR was variable but the PFR was constant, there was not much difference noticeable between the groups, indicating that the change in WRR did not affect the blood removal. For these specific parameters, it can therefore be concluded that WRR over 100% in 20 min (111% for group C) was sufficient. The comparison between Groups D and E (in Figure 5), where the PFR was variable and the WRR was constant, showed a more significant difference, especially for the PL content after seven days and one month of storage. The results of that comparison support that there was more residue blood in Group E, which was bled at a high WRR but which had no pump/PFR. This indicates that with these specific parameters, the PFR had a greater effect on the bleeding efficiency and storage quality of the fillets than the WRR. This is further supported by looking at Group A, which had the highest PFR and lowest WRR, resulting in the highest PL content after seven days and slower formation of FFAs compared to Groups B and C, which bled out in the screw conveyor. Bleeding conditions affected the HI content measured in fresh cod after seven days but evened out to around 0.6–0.7 mg HI/100g after freezer storage (Figure 6). This is in accordance with Minh and Phan [38], where total HI content was initially varied for different bleeding conditions for cobia fish and evened out after 24 weeks of freezer storage. The total HI content in this study was at the same level as the ice water bled cobia studied by Minh and Phan [38] but higher compared to HI in other studies on seabass [39] and tilapia [40], likely due to the different fish species.

The regression analysis shows a significant overall model for six models out of nine, the *R*^2^ values, however, are relatively low (highest at 0.54), indicating that while the WRR and PFR are correlated with the FFA, PL, and HI content, there are other factors explaining some of the variability. The results of the regression model indicate that the variables PFR and WRR have the most significant effects on FFA and PL after one month of freezer storage. For HI content, fewer parameters were found to be significantly different, which is consistent with the results of the ANOVA test. The model showed that more significant parameters were related to the PFR than the WRR. The results of the model also indicate that after four months of freezer storage with temperature fluctuations, there was no difference between the groups in PL results, as there was no significant parameter for PL content. The HI results in Figure 6 indicate a difference between groups for unfrozen cod seven days from catch, as residual blood could act as an oxygen donor effecting PL. After seven days of refrigerated storage, there could also be some effects of residue blood that have yet to emerge in FFA and PL end-point results.

## 5. Conclusions

In this study, the influence of variable pump flow in a bleed-out tank and the rate of seawater recirculation rate during the bleeding process on the changes in FFA, PL, and total HI content in cod muscle was evaluated. Measurements were done on different storage times: on fresh cod seven days after catch, after one month of frozen storage at −24 °C, and, lastly, after four months of simulated frozen food-chain storage (−12 to −18 °C). The results for PL content and the regression model indicate that the enzyme activity in the fish muscle was lower in cases where PFR exerted more influence in the bleeding process than WRR. The effects of successful blood removal also seem to be most noticeable after one month of freezer storage, rather than in fresh cod after seven days or after four months of simulated frozen food-chain storage. The temperature fluctuations during frozen storage for four months resulted in PL values evening out at around 4%, while FFA values were still rising, causing more risk of rancidity in the cod. FFA measurements after four months showed better results for cod bled in tubs compared to the groups bled in a screw conveyor, indicating that higher WRR could have some long-term positive effects. Changes in HI content were less noticeable, as the regression analysis parameters for PFR and WRR were not significant for HI content.

In terms of design parameters for the industry, the study indicates that with the bleeding water to fish ratio of 3:1 and enough WRR (over 100% in 20 min), the PFR becomes the limiting design parameter regarding efficient blood removal and should be at least 10% of the tank volume per minute to ensure enough recirculation and flow of water in the bleed-out tanks.

## Figures and Tables

**Table 1:** Experiment groups settings.

Group	Bleeding Water to Fish Ratio	Pump Flow Recirculation (PFR) (Pump Power)	Water Replacement Frequency	Water Replacement Method	Water Replacement Ratio (WRR) * (Replacement Time)
A	3:1	40 L/s (8 kW)	235 L/min	25 s injection intervals	67% (30 min)
B	3:1	15 L/s (3 kW)	585 L/min	15 s injection intervals	167% (12 min)
C	3:1	15 L/s (3 kW)	390 L/min	10 s injection intervals	111% (18 min)
D	2:1	8 L/s (1.5 kW)	20 L/min	batch replacement	200% (10 min)
E	2:1	No pump	20 L/min	batch replacement	200% (10 min)

*WRR: Ratio of seawater replaced in the bleeding tank/tub during 20 min.

**Table 2:** The regression model estimates. Significant parameter values (with *p* < 0.05) are shown in bold.

Dependent Variables	Parameter/Period	7 Days	1 Month	4 Months
Free Fatty Acids (FFAs)—Equation (2)	b_1_	0.06	**−0.75**	−0.31
	(*p*-value)	(0.36)	(1 × 10^−3^)	(0.15)
	b_2_	2.50	**−11.47**	**−13.04**
	(*p*-value)	(0.13)	(0.03)	(0.02)
	b_0_	3.03	**66.60**	**74.67**
	(*p*-value)	(0.36)	9 × 10^−7^	(4 ×10^−7^)
	*R* ^2^	0.12	0.45	0.24
	Prob(F)	0.17	(3 × 10^−4^)	0.02
Phospholipids (PLs)—Equation (3)	b_1_	**0.14**	**0.16**	−0.01
	(*p*-value)	(3 × 10^−3^)	(3 × 10^−4^)	(0.70)
	b_2_	1.18	**2.17**	0.25
	(*p*-value)	(0.29)	(0.04)	(0.62)
	b_0_	**7.08**	−0.07	3.90
	(*p*-value)	(4 × 10^−3^)	(0.97)	(8 × 10^−4^)
	*R* ^2^	0.53	0.54	0.14
	Prob(F)	4 × 10^−5^	3 × 10^−5^	0.14
Heme Iron (HI)—Equation (4)	b_1_	**0.01**	0.002	−0.002
	(*p*-value)	(0.05)	(0.40)	(0.60)
	b_2_	0.07	−0.06	−0.13
	(*p*-value)	(0.69)	(0.33)	(0.16)
	b_0_	0.53	**0.71**	**0.92**
	(*p*-value)	(0.13)	(5 × 10^−6^)	(4 × 10^−5^)
	*R* ^2^	0.38	0.40	0.17
	Prob(F)	2 × 10^−3^	1 × 10^−3^	0.08
